# Species Diversity of Mycoplankton on the Background of Selected Indicators of Water Quality in Stratified Mesotrophic Lakes

**DOI:** 10.3390/ijerph192013298

**Published:** 2022-10-15

**Authors:** Cudowski Adam, Świsłocka Magdalena

**Affiliations:** 1 Department of Water Ecology, Faculty of Biology, University of Bialystok, Ciolkowskiego 1J, 15-245 Bialystok, Poland; 2 Department of Zoology and Genetics, Faculty of Biology, University of Bialystok, Ciolkowskiego 1J, 15-245 Bialystok, Poland

**Keywords:** mesotrophic lake, species diversity of mycoplankton, water column, physico-chemical properties of water, bacterioplankton, algae

## Abstract

The aim of the study was to determine mycoplankton species diversity in relation to the physico-chemical parameters of lake waters. The research was carried out in the summer months in 15 mesotrophic lakes and showed a high ecological significance index for *Rhodotorula glutinis*, *Epicoccum nigrum*, *Fusarium sporotrichioides*, and *Trichophyton violaceum*. Mycoplankton abundance and species diversity decreased with the depth of water, which coincided with a decrease in oxygen content and organic matter concentration. A high concentration of nitrogen compounds (total nitrogen—TN and dissolved nitrogen—DN) limited the development of mycobiota in the hypolimnion. In the metalimnion, the intensive development of organisms, especially bacteria, limited mycoplankton abundance despite perfect physical and chemical conditions for its development. Finally, mycoplankton functioned the best in slightly alkaline waters.

## 1. Introduction

Fungi are characterized by immense diversity, with the abundance of species ranging from 1.5 to 10 million [1,2,3,4]. Out of around 140,000 fungal species that have been described, only 3000 are found in water [5,6]—primitive fungi (Chytridiomycetes), fungi that have adapted to the aquatic environment (Hyphomycetes, yeasts), or typically terrestrial fungi (various fungi imperfecti, endophytes). In contrast to yeasts which can occur everywhere, aquatic Hyphomycetes and other terrestrial fungi need a solid substrate to survive, and water is only used as a means of transport [7]. In addition, Chytridiomycetes have zoospores adapted for both horizontal and vertical movement within the lake [8], while terrestrial fungi get into the water with wind, rain, and surface run-off [9]. There is some controversy concerning the exact classification of *Aspergillus* or *Penicillium*, genera that are very often isolated in aquatic ecosystems but are considered to be typically terrestrial fungi. On the other hand, there are many fungi found on land whose natural habitat is water [10]. In view of the above, the division of aquatic and terrestrial fungi seems to be problematically ambiguous, so in this paper, we prefer to use the term “mycoplankton,” denoting various fungal microorganisms that can be found in the water column [11].

The main role of fungi occurring in aquatic ecosystems is the decomposition of organic matter [12], which in large quantities, contributes to the increase in lake productivity [13]. According to trophic classification, lakes can be divided into oligotrophic, mesotrophic, and eutrophic. Oligotrophic lakes are deep lakes with crystal-clear water, where the bottom is sandy and poor in both plants and animals. Eutrophic lakes, on the other hand, are characterized by the brown coloration of the water, muddy bottoms, and, very often, anaerobic hypolimnia. These waters are often subject to intensive, uncontrolled algal growth. Mesotrophic lakes, with moderate levels of organic substances and nutrients [14], represent a transition stage between oligotrophy and eutrophy, with a loss of balance between production, consumption, and decomposition of organic matter [15], leading to the deterioration of the trophic status of waters [16].

Considering the role of mycobiota in the biotransformation of organic matter, it can be assumed that these organisms may also take part in limiting the eutrophication of water reservoirs. Therefore, the aim of this study was to determine mycoplankton species diversity in the profiles of mesotrophic lakes. Another aim of the study was to determine the relationship between the physico-chemical parameters of water, algae, and bacteria and the mycobiota of the mesotrophic lakes studied. Finally, the intention was also to determine the mycoplankton species with the highest index of ecological significance in the studied lake ecosystems. The research also made it possible to improve the knowledge of the ecology of aquatic fungi and, in particular, to learn about the most important factors determining the species composition and the number of water fungi in mesotrophic lakes.

## 2. Material and Methods

### 2.1. Field Study

Our research was conducted on 15 mesotrophic lakes, 5 of which were characterized by aerobic conditions in the entire profile, 5 by lack of oxygen only in the hypolimnion and 5 by limited access to oxygen only in the hypolimnion (Table 1). The period of sampling was characterized by convenient meteorological conditions for limnological studies and ensured that reliable data was obtained.

The data were collected during the peak of summer. All the lakes were non-flow-through lakes or lakes with a very limited inflow of river waters. The lakes were located in north-eastern Poland, central Europe. Water samples were taken with a Limnos bathymeter from three layers (epilimnion, metalimnion, and hypolimnion). A representative sample for each layer was a mixture of incremental 1 L samples taken every 1 m. After the water had been sampled, the representative samples were stored in sterile containers in a refrigerator at 5 °C until they were transported to the laboratory for further analysis.

### 2.2. Hydrochemical Laboratory Tests

In the field, water temperature, pH, electrolytic conductivity, dissolved oxygen (DO), and saturated dissolved oxygen (SDO) were measured using Hach Lange probes. The concentrations of total organic carbon (TOC), dissolved organic carbon (DOC), and inorganic carbon (IC), as well as total nitrogen (TN) and dissolved nitrogen (DN) were determined in the laboratory using a high-temperature catalytic combustion method in a Shimadzu TOC-5050A analyzer (Kyoto, Japan).

### 2.3. Microbiological Laboratory Tests

The algae biomass was measured by the submersible spectrofluorometer (FluoroProbe, bbe-Moldaenke, Schwentinental, Germany). The total bacterial abundance was determined by incubation on a nutrient agar according to PN-EN ISO 6222. Water samples were plated on nutrient agar (Biocorp, Issoire, France). The plates were incubated for 68 h at 22 °C, and after this time, all colonies grown on the plate were counted. The result was expressed in CFU/mL.

In order to cultivate fungal colonies, 250 mL samples of untreated water diluted 1:100 were collected. Then each sample was inoculated directly into three types of Petri dishes containing: Sabouraud Dextrose with chloramphenicol and gentamicin (Biocorp), Potato Dextrose (Biocorp), and Malt Extract Biocorp. The plates prepared in this way were incubated at 36 °C and 25 °C (due to differences in optimal growth temperature) for 5 days. After the end of the incubation of the plates containing Sabouraud’s medium, the total abundance of colonies was determined [17]. DNA for molecular analysis was isolated from colonies grown on all media. Colonies after 5 days of incubation were collected with 1.5 mL Eppendorf tubes, and the DNA was isolated using the Genomic Mini AX Yeast and Bead-Beat Micro Gravity DNA Isolation Kit according to the instructions included in the kit [18]. This material was used for molecular studies, using amplifying (PCR) with a genomic DNA fragment with an average length of 600 pz, which is characteristic of the whole fungal kingdom. PCR amplification of ITS1 was carried out with a GeneAmp PCR System 9700 (Foster City, CA, USA) in 5 μL volumes, and the reaction mixtures consisted of 2 μL of extracted genomic DNA as a template, 1.7 μL of Qiagen Multiplex PCR Master Mix (1×), 0.3 μL mix primers and 1 μL of Qiagen nuclease-free water.

Universal primers were used to perform the PCR reaction: ITS1 (5′-CTTGGTCATTTAGAGGAAGTAA-3′), which contains sequences complementary to the 18S gene section at the 3′ end, and ITS4 (5′-TCCTCCGCTTATTGATATGC-3′), which is complementary to the 28S region [19]. The products of this reaction were purified with the EPPiC Fast mix (A&A Biotechnology, Gdańsk, Poland) in an enzymatic reaction following the manufacturer’s protocols. They were then processed for cycle sequencing PCR with a BigDye™ Terminator v3.1 Cycle Sequencing Kit v.3.1 (Applied Biosystems, Foster City, CA, USA) using primer forward (ITS_1). Unincorporated dideoxynucleotides were eliminated from the sequencing reaction using the ExTerminator Kit (A&A Biotechnology). In the last stage of the research, a fragment of ITS was directly analyzed in an automatic sequencer (Applied Biosystems), and then we used software. The obtained sequences were read, matched, aligned, and then compared to the sequences of standard strains deposited in the NCBI—GenBank database using the BLAST application, which determined the species composition of mycoplankton in the profiles of the dimictic mesotrophic lakes [20]. The query coverage [in %] demonstrates the percentage of the query sequence length that is included in the alignment of analyzed all sequences compared to the haplotypes available from GenBank was 100%. Percent identity, which is calculated as the % of characters within the covered part of the query that is identical for those obtained in our study sequences compared to haplotypes available from GenBank, ranged from 98.21 (*Heliscus lugdunensis*) to 100% (for 75% recognized fungi species)—Table 2.

### 2.4. Ecological Indicators and Statistical Analyses

Kasprzak and Niedbała’s ecological significance index (Q) was calculated as the quotient of the product of dominance and frequency expressed as percentages [21]. The value of Q ≥ 30% was assumed to be very high, while the value of Qє (15–30%) was assumed to be high. The ecological significance index was calculated based on the formula:

Q = D·F/100

Q—ecological significance index
F—frequency
D—domination

Relationships between nominal and quantitative variables were calculated with Cramér’s V, but relationships between quantitative variables were calculated with Pearson’s correlation coefficient (r). For the interpretation of the relationship between the environmental and biological data, redundancy analysis (RDA) was performed. To test whether the RDA analysis was appropriate for the dataset, the data were previously tested for normality (Kolmogorov–Smirnov test). Detrended correspondence analysis (DCA) was used first to determine the character of variability in the studied communities: if the length of the first gradient was greater than 2 standard deviations, we assumed a unimodal variation; a length smaller than 2 SD indicated a linear variation [22]. The length of the first gradient for the fungal communities amounted to 1.71 SD, which indicated a linear variation, providing justification for the further use of the redundancy analysis. Statistical calculations were performed with the Statistica v. 13.3 software.

## 3. Results

### 3.1. Measurements of Physicochemical Parameters of Water

The lakes were characterized by a moderate electrolytic conductivity of 380 μS/cm, with the lowest mean conductivity in the epilimnion and the highest in the hypolimnion (Table 3). Similar dependencies were observed for the concentrations of total inorganic carbon and total and dissolved nitrogen, i.e., with an increase in depth, the concentrations of these parameters increased, with their average levels being 44.0 mgC/L, 1.26 μgN/L, and 1.08 μgN/L, respectively (Table 3). The examined lakes were alkaline in character with an average of pH = 8.03 and relatively good oxygenation. The average concentration of oxygen in the waters was 6.48 mg/L, and the average oxygen saturation was 70%. In addition, the average concentration of total organic carbon was 6.13 mgC/L, while that of dissolved organic carbon was 5.71 mgC/L. It should be noted that the average concentrations of these parameters decreased with depth (Table 3). The average algae biomass in the mesotrophic lakes studied was 5.78 μg/L, with the highest density found in the metalimnion and the lowest in the hypolimnion (Table 3). Algae biomass in the hypolimnion of the studied lakes differed significantly from algae biomass in the epilimnions and metalimnions of the examined waters, with a level of significance at *p* < 0.001. A similar observation was made for bacterioplankton, where the average abundance of cells/mL was 2,420,000, and the difference in the abundance of bacteria between the hypolimnion and the two other layers was statistically significant at *p* < 0.005 (Table 3).

### 3.2. PCR/Sequencing Analysis

In the mesotrophic lakes studied, DNA sequencing results allowed for the identification of 55 species of fungi—36 species in the epilimnion, 34 in the metalimnion, and 24 in the hypolimnion (Table 4). The average abundance of fungi was 9800 CFU/mL, with the highest abundance in the epilimnion and the lowest in the hypolimnion (Table 3).

### 3.3. Relationship between Water Quality and Mycoplankton

A statistically significant correlation was found between the abundance of mycoplankton and TOC (r = 0.81, *p* < 0.05). The abundance of mycoplankton in the hypolimnion of mesotrophic lakes differed significantly from the abundance of fungi in the epilimnion (*p* < 0.001) or metalimnion (*p* < 0.05). All lakes were dominated by Ascomycota and Dothidomycetes fungi.

Among the individual groups of mesotrophic lakes, the highest species diversity was found in the waters with aerobic hypolimnion, where 12 species of fungi were found on average, and the lowest in waters with anaerobic hypolimnion, where on average four species of fungi were found.

**Figure 1:**
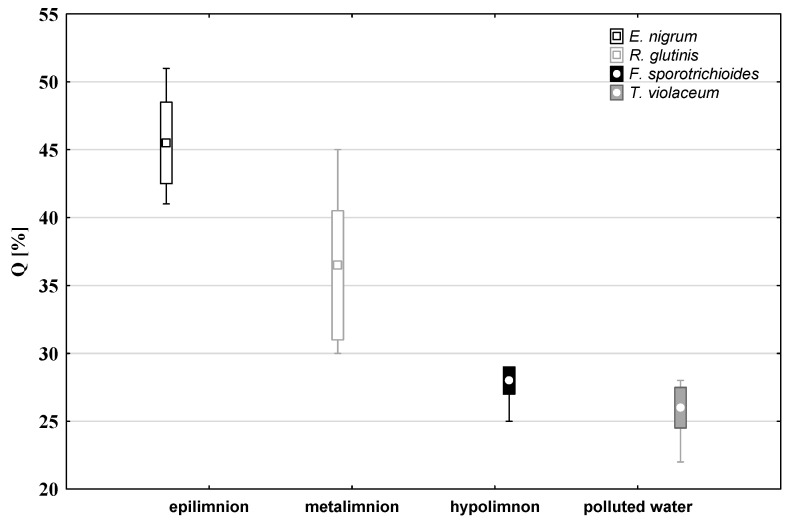
Values of the indicator of ecological significance (Q) of select mycoplankton species.

Two species were found in all the lakes in all their layers: *Epicoccum nigrum* and *Rhodotula glutinis* (Table 4). *Rhodotorula glutinis* (Figure 1) showed a very high ecological significance index in the metalimnion and *Epicoccum nigrum* (Figure 1) in the epilimnion.

**Figure 2:**
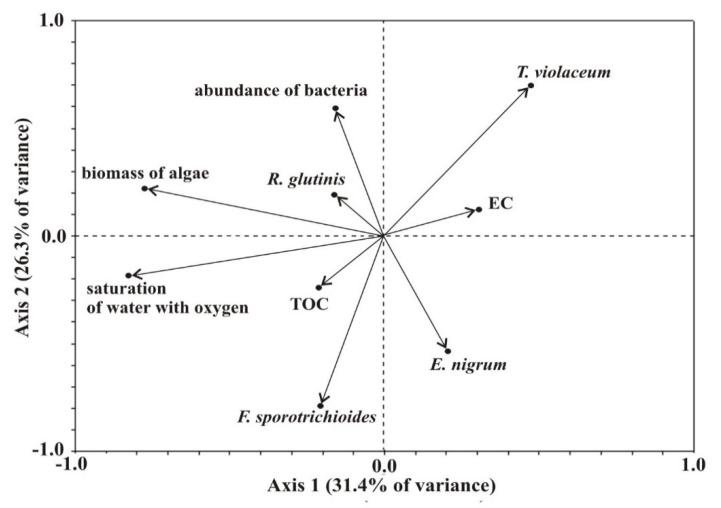
Diplot of redundancy analysis (RDA) results evaluating the relationships between the four species of mycoplankton characterized by the significant ecological importance and environmental variables. Environmental variables and fungal species are shown as arrows. The vector orientations represent the direction of the greatest change; vector lengths represent relative importance, while perpendicular arrows indicate no correlation.

*Rhodotorula glutinis* showed a directly proportional relationship between its abundance and algae biomass and the abundance of bacteria. An inversely proportional relationship was observed for *Epicoccum nigrum* (Figure 2).

*Pleosporaceae* sp. and *Fusarium sporotrichioides* were found exclusively in the hypolimnions of lakes with entirely aerobic profiles, the latter showing a high ecological significance index (Figure 1). *Cladosporium herbarum* was present in each layer, except for the hypolimnions of the studied lakes. In turn, *Trichophyton violaceum* was found exclusively in the layers of the lake where the electrolytic conductivity exceeded 500 μS/cm, i.e., those with considerable mineral water pollution; the species had a high ecological significance index (Figure 1).

*Naganishia albida* was a species specific to lake layers with an alkaline pH (pH > 9), and the total abundance of fungi was much lower than in the other layers. Species that appeared only in the surface layer included *Pithomyces chartarum*, *Naganishia diffluens*, and *Naganishia alba*, albeit *Naganishia diffluens* appeared only in the epilimnions of lakes with anaerobic hypolimnions (Table 4).

In the case of the lakes studied, it was observed that the species diversity of fungi decreased by over 50% on average between the surface and the bottom, which positively correlated with the DOC (r = 0.78, *p* < 0.005). However, the exception to this rule was the group of lakes with aerobic conditions in their entire profiles (Table 4). The hypolimnion was the layer characterized by the highest repeatability of species within this group of lakes.

The study showed a statistically significant difference (*p* < 0.005) in the species diversity of mycoplankton between the epilimnion and hypolimnion in the lakes characterized by a lack of oxygen in the hypolimnion. There was a statistically significant correlation between mycoplankton abundance and water saturation with oxygen (r = 0.87, *p* < 0.001).

## 4. Discussion

Although fungi are important in the carbon and nutrient cycle of aquatic ecosystems, our knowledge of their specific role is limited [23,24,25]. According to the literature, humic substances, such as humic acid or fulvic acids, have a protective function against stressful environmental factors. They induce the synthesis of stress proteins, including chaperones and heat shock proteins, and they transform xenobiotics and poisons and activate antioxidant enzymes, thus improving the living conditions of organisms [26]. On the other hand, at high concentrations, humic substances can act as stressors, causing lipid peroxidation, and are even able to induce mechanisms of carcinogenesis [26]. It should be noted, however, that the effect of their impact on living organisms depends on their concentration and internal structure, which in turn depend on their source in the natural environment [26].

Because mesotrophic lakes are characterized by medium fertility and moderately high biological production, it can be assumed that they are a good environment for mycoplankton. At low concentrations, humic substances stimulate bacteria and algae growth [26]. As shown by the results of our study, the abundance and species diversity of aquatic fungi decreased with a decrease in the concentration of organic matter. The decrease may also have been caused by deteriorating oxygen conditions at greater depths. Moreover, the lower species diversity of mycobiota could also have resulted from the fact that nitrogen concentrations in the hypolimnion were much higher than in other lake layers. The negative effect of high concentrations of nitrates (V) or ammonium nitrogen on the development of water fungi was also noted by Godlewska et al. [27]. They claimed that increased concentrations of nitrogen or organic carbon limited the development of mycobiota. Muszyńska et al. [28], on the other hand, state that too low concentrations of nutrients in water limit the development of planktonic fungi. However, some types of fungi, such as *Aspergillus*, *Mucor*, *Rhizopusand*, and *Penicillium*, can thrive in conditions with limited access to organic matter because they can produce organic water-soluble acids themselves [29,30].

Available research results clearly indicate a complex relationship between mycoplankton and organic matter determining the progress of water eutrophication [31] together with other biogens, e.g., nitrogen, phosphorus [32], or manganese [33]. However, large amounts of humic substances reduce light availability, thus inhibiting photosynthesis (blocking photosystem II) and chlorophyll synthesis, which in turn leads to the inhibition of algal growth [26]. Intensive algae development, resulting from progressing water eutrophication, leads to the deterioration of oxygen conditions, which play a very important role in fungal development [34]. Therefore, in those parts of the lake where an oxygen deficit exists, the abundance and species diversity of mycoplankton were much smaller than in waters with significant oxygen content. This does not mean, however, that fungi occur only sporadically in such conditions. There are also many fungal species that are actively involved in water treatment [35], thus regulating increases in the productivity of water reservoirs.

It might seem that another factor influencing the development of aquatic fungi is pH, but literature reports present highly discrepant results. Mazurkiewicz-Zapałowicz et al. [36] claim that the greatest diversity of mycobiota occurs at a slightly alkaline water pH, which is confirmed by Ortiz-Vera et al. [37], who found the development of pathogenic fungi to be dependent on an alkaline environment. Similarly, consider Baudoin et al. [38], who observed a decrease in fungal species diversity with a decrease in pH. In contrast, Wurzbacher et al. [7] indicate that mycobiota prefer an acidic environment, while in a paper by Pietryczuk et al. [39], the development of aquatic hyphomycetes did not depend on water pH. This was echoed by Lima et al. [40], who point to factors other than water pH in determining the development of fungi.

In this study, the abundance and species diversity of mycobiota decreased with increasing acidity. However, it should be noted that the examined waters, despite the decrease in pH, were still alkaline (pH > 7). The alkaline character of the examined lake waters did not limit the development of mycoplankton, and this environment also favors the development of bacteria [41,42]. Nevertheless, fungi and bacteria cannot develop side by side under favorable pH conditions, both in terms of their abundance [43] and their species composition [44]. As shown by the Mille-Lindblom and Tranvik [45] studies, the development of bacterial biomass significantly hindered the development of water fungi, which is probably why in our study, the abundance of mycobiota decreased in the metalimnion, despite the excellent conditions for their development (optimal content of carbon, nitrogen, slightly alkaline pH, and good oxygenation of water). Moreover, in this layer, there is an intensive development of zooplankton [46], which feeds, among others, on mycoplankton [47].

Among all the identified species of water fungi in the studied lakes, only four species were rated at the highest level of ecological significance. One of them was *Rhodotorula glutinis*, a fungus that has been isolated from extreme aquatic environments such as deep reservoirs, high-temperature waters, or areas polluted by domestic wastewater [48]. This may explain the presence of this species in the metalimnion of the examined waters, a layer with incomparably higher bacterial and algal abundance compared to the other layers. According to Kot et al. [49], *Rhodotorula glutinis* may produce numerous organic compounds, i.e., lipids or enzymes, but it may also use several organic compounds as a source of carbon, hence the presence of this species in the layer characterized by a great wealth of living organisms, such as zooplankton or rotifers [50].

*Epicoccum nigrum* was another fungus with a very high ecological significance index in the epilimnion. Its occurrence in this layer was connected to its ability to colonize some algal species [51], whose biomass was relatively high. Additionally, there was no intensive bacterial development in this layer. This is particularly important because, according to many authors, this fungus produces numerous antibacterial compounds [52,53], so the existence of bacterioplankton near this fungus is limited. Significantly, this fungus has been detected in the air, from where it is able to enter the surface layer of water without any great restrictions [54,55]. Moreover, it is also an example of a microorganism that colonizes various soil types and plant species [56], which may facilitate its transport into the surface layers of lakes with run-off.

*Fusarium sporotrichioides* were characterized by a high ecological significance index in the hypolimnions of the mesotrophic lakes with good oxygen conditions in their entire profiles. Fungi from this family are very sensitive to weather conditions [57]. Therefore, it was likely this species was present in the layer that was least exposed to weather conditions. In addition, this fungus grows on living and dead organisms, so it can fall to the bottom of the reservoir together with the dead remains [58] and exist there in convenient conditions. Moreover, *Fusarium sporotrichioides* can get into the hypolimnion on bread fed by people to birds and fish because this fungus is a parasite of wheat [59].

Another fungus, *Trichophyton violaceum*, was not specific to any layer of the mesotrophic lakes under study but was related to the degree of pollution—a high ecological significance index for this microorganism was recorded in the layers where EC > 500 mS/cm. This fungus is a dermatophyte that uses keratin as a source of carbon [60,61] and can enter into the epilimnion or metalimnion with the epidermis and skin appendages. Moreover, as Shekha et al. [62] suggested, this fungus occurs in the bottom sediments of heavily polluted waters, which could explain its presence in the hypolimnion of the examined waters.

## 5. Conclusions

The abundance and taxonomic structure of mycoplankton in the mesotrophic lake profiles depended mainly on the saturation of water with oxygen and the concentration of organic matter. It was shown that the abundance and abundance of mycoplankton species decreased with the depth of the lake, which coincided with the decrease in oxygen content and organic matter concentration in the profiles of the studied aquatic ecosystems.

An additional factor limiting the development of fungi in the bottom layers of mesotrophic lakes was the high concentration of nitrogen compounds.

The acidity of the water is also significant for the development of mycobiota. As shown by the test results, a slightly alkaline pH was optimal for the development of this group of microorganisms.

Moreover, it was shown that the metalimnions of mesotrophic lakes were characterized by a much smaller abundance of mycoplankton in comparison to the epilimnions, despite the excellent physical and chemical conditions for their development. This was mainly due to the fact that the metalimnions of mesotrophic waters were marked by a huge abundance of bacterioplankton, which demonstrates antagonistic interactions with mycobiota.

The results of the study also showed that in the studied mesotrophic lakes, only four species of fungi were important from the point of view of ecological significance. *Rhodotorula glutinis* was of particular ecological importance in the metalimnion, *Epicoccum nigrum* in the epilimnion, and *Fusarium sporotrichioides* in the hypolimnion of lakes with aerobic conditions throughout their entire profiles. *Trichophyton violaceum* had a high ecological index in water waters contaminated with dissolved substances.

## Figures and Tables

**Table 1:** Water sampling sites with geographic coordinates.

Oxygen Conditions in Lake	Lake	Geographical Coordinates
The presence of oxygen in the entire profile	Szurpiły	54.22746991 N, 22.89284831 E
	Staw	54.02284682 N, 22.98780206 E
	Ożewo	54.14730288 N, 22.81103507 E
	Białe Wigierskie	54.03053768 N, 23.08963512 E
	Czarne near Bryzgla	54.00539434 N, 23.08847648 E
Anaerobic hypolimnion	Jeglówek	54.23828083 N, 22.88764166 E
	Jaczno	54.28019417 N, 22.87475052 E
	Kojle	54.27619198 N, 22.89210078 E
	Perty	54.27352605 N, 22.89788832 E
	Dusajtys	54.14202472 N, 23.42812649 E
Limited oxygen in the hypolimnion	Ryngis	54.09177329 N, 23.43347970 E
	Muliczne	54.02806960 N, 23.03853835 E
	Hałny	54.13625607 N, 23.45802686 E
	Pobłędzie	54.30814827 N, 22.75552361 E
	Sztabinki	54.12678003 N, 23.41988603 E

**Table 2:** List of fungal species and GenBank accession numbers of their sequences used in our molecular analysis. Query coverage [in %] demonstrates the percentage of the query sequence length that is included in the alignment. Percent identity is calculated as the % of characters within the covered part of the query that is identical.

Species Name	GenBank Acc. Num.	Query Coverage/ Percent Identity [in %]	Data Availability
*Acremonium implicatum/* *Sarocladium implicatum*	MT635273	100/100	https://www.ncbi.nlm.nih.gov/nuccore/MT635273 (accessed on 16 June 2020)
*Alternaria alternata*	MT635274	100/99.82	https://www.ncbi.nlm.nih.gov/nuccore/MT635274 (accessed on 16 June 2020)
*Alternaria brassicicola*	MT635275	100/100	https://www.ncbi.nlm.nih.gov/nuccore/MT635275 (accessed on 16 June 2020)
*Alternaria infectoria*/ *Lawia infectoria*	MT635276	100/99.83	https://www.ncbi.nlm.nih.gov/nuccore/MT635276 (accessed on 16 June 2020)
*Anguillospora crassa*	MT635277	100/100	https://www.ncbi.nlm.nih.gov/nuccore/MT635277 (accessed on 16 June 2020)
*Uncultured Ascomycota/* *Ascomycota* sp.	MT635278	100/100	https://www.ncbi.nlm.nih.gov/nuccore/MT635278 (accessed on 16 June 2020)
*Aspergillus fumigatus*	MT635279	100/100	https://www.ncbi.nlm.nih.gov/nuccore/MT635279 (accessed on 16 June 2020)
*Aspergillus tabacinus*	MT635280	100/100	https://www.ncbi.nlm.nih.gov/nuccore/MT635280 (accessed on 16 June 2020)
*Aspergillus westerdijkiae*	MT635281	100/100	https://www.ncbi.nlm.nih.gov/nuccore/MT635281 (accessed on 16 June 2020)
*Bipolaris sorokiniana*/ *Cochliobolus sativus*	MT635282	100/100	https://www.ncbi.nlm.nih.gov/nuccore/MT635282 (accessed on 16 June 2020)
*Bjerkandera adusta*	MT635283	100/99.84	https://www.ncbi.nlm.nih.gov/nuccore/MT635283 (accessed on 16 June 2020)
*Cadophora fastigiata*	MT635284	100/100	https://www.ncbi.nlm.nih.gov/nuccore/MT635284 (accessed on 16 June 2020)
*Candida albicans*	MT635285	100/100	https://www.ncbi.nlm.nih.gov/nuccore/MT635285 (accessed on 16 June 2020)
*Cladosporium cladosporioides*	MT635286	100/100	https://www.ncbi.nlm.nih.gov/nuccore/MT635286 (accessed on 16 June 2020)
*Cladosporium halotolerans*	MT635287	100/100	https://www.ncbi.nlm.nih.gov/nuccore/MT635287 (accessed on 16 June 2020)
*Cladosporium herbarum/* *Mycosphaerella tassiana*	MT635288	100/99.81	https://www.ncbi.nlm.nih.gov/nuccore/MT635288 (accessed on 16 June 2020)
*Davidiella* sp.	MT635289	100/100	https://www.ncbi.nlm.nih.gov/nuccore/MT635289 (accessed on 16 June 2020)
*Epicoccum nigrum*	MT635290	100/100	https://www.ncbi.nlm.nih.gov/nuccore/MT635290 (accessed on 16 June 2020)
*Exophiala xenobiotica*	MT635291	100/100	https://www.ncbi.nlm.nih.gov/nuccore/MT635291 (accessed on 16 June 2020)
*Filobasidium magnum*	MT635292	100/100	https://www.ncbi.nlm.nih.gov/nuccore/MT635292 (accessed on 16 June 2020)
*Flagellospora curvula*	MT635293	100/100	https://www.ncbi.nlm.nih.gov/nuccore/MT635293 (accessed on 16 June 2020)
*Fomitopsis pinicola*	MT635294	100/100	https://www.ncbi.nlm.nih.gov/nuccore/MT635294 (accessed on 16 June 2020)
*Fusarium acuminatum*	MT635295	100/100	https://www.ncbi.nlm.nih.gov/nuccore/MT635295 (accessed on 16 June 2020)
*Fusarium equiseti/* *Gibberella intricans*	MT635296	100/100	https://www.ncbi.nlm.nih.gov/nuccore/MT635296 (accessed on 16 June 2020)
*Fusarium poae*	MT635297	100/100	https://www.ncbi.nlm.nih.gov/nuccore/MT635297 (accessed on 16 June 2020)
*Fusarium sporotrichioides*	MT635298	100/100	https://www.ncbi.nlm.nih.gov/nuccore/MT635298 (accessed on 16 June 2020)
*Hanseniaspora uvarum*	MT635299	100/100	https://www.ncbi.nlm.nih.gov/nuccore/MT635299 (accessed on 16 June 2020)
*Glonium pusillum*	MT635300	100/99.81	https://www.ncbi.nlm.nih.gov/nuccore/MT635300 (accessed on 16 June 2020)
*Heliscus lugdunensis*/ *Neonectria lugdunensis*	MT635301	100/98.21	https://www.ncbi.nlm.nih.gov/nuccore/MT635301 (accessed on 16 June 2020)
*Holtermanniella takashimae*	MT635302	100/100	https://www.ncbi.nlm.nih.gov/nuccore/MT635302 (accessed on 16 June 2020)
*Komagataella phaffii*	MT635303	100/99.63	https://www.ncbi.nlm.nih.gov/nuccore/MT635303 (accessed on 16 June 2020)
*Lunulospora curvula*	OP444247	100/100	https://www.ncbi.nlm.nih.gov/nuccore/OP444247 (accessed on 14 September 2022)
*Meyerozyma guilliermondii*	OP444248	100/100	https://www.ncbi.nlm.nih.gov/nuccore/OP444248 (accessed on 14 September 2022)
*Microdochium* sp.	OP444249	100/99.63	https://www.ncbi.nlm.nih.gov/nuccore/OP444249 (accessed on 14 September 2022)
*Naganishia albida*	OP444250	100/100	https://www.ncbi.nlm.nih.gov/nuccore/OP444250 (accessed on 14 September 2022)
*Naganishia diffluens*	OP444251	100/100	https://www.ncbi.nlm.nih.gov/nuccore/OP444251 (accessed on 14 September 2022)
*Penicillium chrysogenum*	OP444252	100/100	https://www.ncbi.nlm.nih.gov/nuccore/OP444252 (accessed on 14 September 2022)
*Penicillium griseoroseum*	OP444253	100/99.83	https://www.ncbi.nlm.nih.gov/nuccore/OP444253 (accessed on 14 September 2022)
*Penicillium olsonii*	OP444254	100/100	https://www.ncbi.nlm.nih.gov/nuccore/OP444254 (accessed on 14 September 2022)
*Didymella pinodella*	OP444255	100/100	https://www.ncbi.nlm.nih.gov/nuccore/OP444255 (accessed on 14 September 2022)
*Pichia fermentans*/ *Candida lambica*	OP444256	100/99.50	https://www.ncbi.nlm.nih.gov/nuccore/OP444256 (accessed on 14 September 2022)
*Pichia guilliermondii*	MT635314	100/100	https://www.ncbi.nlm.nih.gov/nuccore/MT635314 (accessed on 16 June 2020)
*Pithomyces chartarum*	MT635315	100/100	https://www.ncbi.nlm.nih.gov/nuccore/MT635315 (accessed on 16 June 2020)
*Pleosporaceae* sp.	MT635316	100/100	https://www.ncbi.nlm.nih.gov/nuccore/MT635316 (accessed on 16 June 2020)
*Pseudozyma pruni*	MT635317	100/99.63	https://www.ncbi.nlm.nih.gov/nuccore/MT635317 (accessed on 16 June 2020)
*Rhodotorula glutinis*	MT635318	100/99.83	https://www.ncbi.nlm.nih.gov/nuccore/MT635318 (accessed on 16 June 2020)
*Rhodotorula mucilaginosa*	MT635319	100/99.83	https://www.ncbi.nlm.nih.gov/nuccore/MT635319 (accessed on 16 June 2020)
*Simplicillium* sp.	MT635320	100/100	https://www.ncbi.nlm.nih.gov/nuccore/MT635320 (accessed on 16 June 2020)
*Talaromyces purpureogenus*	MT635321	100/100	https://www.ncbi.nlm.nih.gov/nuccore/MT635321 (accessed on 16 June 2020)
*Trametes hirsuta*	MT635322	100/99.84	https://www.ncbi.nlm.nih.gov/nuccore/MT635322 (accessed on 16 June 2020)
*Trichoderma harzianum*	MT635323	100/100	https://www.ncbi.nlm.nih.gov/nuccore/MT635323 (accessed on 16 June 2020)
*Trichophyton violaceum*	MT635324	100/100	https://www.ncbi.nlm.nih.gov/nuccore/MT635324 (accessed on 16 June 2020)
*Uncultured fungus*	MT635325	100/100	https://www.ncbi.nlm.nih.gov/nuccore/MT635325 (accessed on 16 June 2020)
*Uncultured Nectriaceae*	MT635326	100/100	https://www.ncbi.nlm.nih.gov/nuccore/MT635326 (accessed on 16 June 2020)
*Ustilaginoidea virens*/ *Villoscislava virens*	MT635327	100/100	https://www.ncbi.nlm.nih.gov/nuccore/MT635327 (accessed on 16 June 2020)

**Table 3:** Average values and standard deviations of selected physicochemical parameters of water quality and the abundance of bacteria and fungi for particular layers of mesotrophic lakes.

Parameter [Unit]	The Presence of Oxygen in the Entire Profile			Anaerobic Hypolimnion			Limited Oxygen in Hypolimnion		
Layer	E	M	H	E	M	H	E	M	H
**EC [μS/cm]**	382 ± 39.6	446 ± 40.2	478 ± 37.7	400 ± 31.8	414 ± 35.1	453 ± 38.5	289 ± 82.6	317 ± 82.1	329 ± 83.5
**pH**	8.37 ± 0.61	8.12 ± 0.36	7.62 ± 0.74	8.05 ± 0.08	7.91 ± 0.15	7.07 ± 0.19	8.42 ± 1.05	8.08 ± 0.16	7.83 ± 0.37
**DO [mg/L]**	11.2 ± 3.33	13.0 ± 0.62	7.14 ± 1.10	8.35 ± 0.44	10.3 ± 2.36	0.20 ± 0.05	8.69 ± 0.83	5.46 ± 0.73	3.55 ± 0.15
**SDO [%]**	131 ± 21.7	121 ± 23.1	64.9 ± 6.01	116 ± 15.5	107 ± 22.3	2.3 ± 1.7	103 ± 12.4	44.3 ± 7.02	28.6 ± 1.34
**TOC [mgC/L]**	5.97 ± 1.82	5.00 ± 0.92	4.31 ± 1.09	6.66 ± 1.61	5.77 ± 1.50	5.34 ± 1.45	7.30 ± 1.93	6.59 ± 1.85	6.16 ± 1.67
**DOC [mgC/L]**	5.18 ± 2.61	4.75 ± 0.92	3.99 ± 1.24	5.95 ± 2.11	5.48 ± 1.40	5.00 ± 1.52	6.91 ± 1.91	6.24 ± 1.81	6.04 ± 1.60
**IC [mgC/L]**	39.5 ± 3.42	47.9 ± 5.92	53.4 ± 7.97	48.0 ± 3.21	51.0 ± 4.33	58.0 ± 4.54	31.5 ± 8.33	35.5 ± 8.36	37.2 ± 8.67
**TN [mgN/L]**	1.24 ± 0.28	1.83 ± 0.48	1.99 ± 0.85	1.18 ± 0.12	1.53 ± 0.39	2.15 ± 0.47	0.79 ± 0.08	0.91 ± 0.25	1.37 ± 0.49
**DN [mgN/L]**	1.08 ± 0.23	1.69 ± 0.52	1.81 ± 0.95	0.94 ± 0.17	1.32 ± 0.19	1.81 ± 0.34	0.69 ± 0.04	0.83 ± 0.21	1.31 ± 0.51
**Biomass of algae [μg/L]**	6.68 ± 0.87	8.76 ± 1.05	2.76 ± 0.09	5.02 ± 0.37	6.04 ± 0.42	0.89 ± 0.01	5.97 ± 0.88	7.09 ± 0.91	1.94 ± 0.11
**Abundance of bacteria 10^6^ [cells/mL]**	1.8 ± 0.7	4.6 ± 1.1	0.7 ± 0.5	1.7 ± 0.6	4.1 ± 1.2	1.3 ± 0.7	1.7 ± 0.9	4.2 ± 1.3	0.6 ± 0.3
**Abundance of fungi [CFU/mL]**	11,800 ± 3800	9000 ± 2600	5600 ± 2200	11,000 ± 4600	6600 ± 1400	4400 ± 3000	11,400 ± 4200	7800 ± 1800	4800 ± 1800

**Table 4:** Species diversity of mycoplankton in mesotrophic lake profiles depending on oxygen availability. X—species appearing in 5 out of 5 examined lakes, x—species appearing in 4 out of 5 examined lakes, ˅—species appearing 3 out of 5 lakes, ˄—species appearing 1 out of 5 lakes studied.

The Presence of Oxygen in the Entire Profile	Layer			Anaerobic Hypolimnion	Layer			Limited Oxygen in Hypolimnion	Layer		
	E	M	H		E	M	H		E	M	H
*Acremonium implicatum*	X		X	*Alternaria alternata*	X	˅		*Alternaria alternata*	X		
*Anguillospora crassa*	x	X		*Alternaria infectoria*		X		*Cladosporium cladosporioides*	X	X	X
*Ascomycota clone*	X			*Alterniaria brassicicola*		X		*Cladosporium halotolerans*	X		
*Aspergillus fumigatus*		X	˅	*Anguillospora crassa*	X	x		*Cladosporium herbarum*	X	X	
*Bjerkandera adusta*	X	X	X	*Aspergillus fumigatus*		X		*Epicoccum nigrum*	X	X	X
*Cladosporium cladosporioides*			X	*Aspergillus tabacinus*	X			*Exophiala xenobiotica*	X		
*Cladosporium halotolerans*	x	X		*Aspergillus westardijkiae*			X	*Holtermanniella takashimae*			X
*Cladosporium herbarum*	x			*Bipolaris sorokiniana*	X			*Meyerozyma guilliermondii*	X	X	
*Davidiella* sp.	x		X	*Cadophora fastigiata*		X		*Naganishia albida*	˅		
*Epicoccum nigrum*	X	X	X	*Candida albicans*		˅		*Penicillium chrysogenum*	X	x	X
*Flagellospora curvula*	X	X	X	*Cladosporium cladosporioides*	X	X	X	*Penicillium olsonii*	x		
*Fusarium sporotrichioides*			X	*Cladosporium halotolerans*	X	X	X	*Phoma pinodella*		X	X
*Glonium pusillum*		x		*Cladosporium herbarum*	X			*Pichia guilliermondii*	X	X	
*Heliscus lugdunensis*	X	X	X	*Epicoccum nigrum*	X	X	X	*Pithomyces chartarum*	X		
*Komagataella phaffii*		˅		*Filobasidium magnum*		X		*Pseudozyma pruni*	x		X
*Lunulospora curvula*	X	X		*Fomitopsis pinicola*		X		*Rhodotorula glutinis*	X	X	X
*Meyerozyma guilliermondii*	X		X	*Fusarium acuminatum*	X			*Rhodotorula mucilaginosa*	X	X	X
*Penicillium chrysogenum*	x	X	x	*Fusarium equiseti*	X			*Simplicillium* sp.	x		
*Pichia guilliermondii*	X	˅	X	*Fusarium poae*			x	*Talaromyces purpureogenus*		X	X
*Pleosporaceae* sp.			X	*Hanseniaspora uvarum*		˄		*Ustilaginoidea virens*	x	˄	
*Rhodotorula glutinis*	X	X	X	*Heliscus lugdunensis*	x		x				
*Talaromyces purpureogenus*		X	X	*Holtermanniella takashimae*			X				
*Trichoderma harzianum*	X		X	*Komagataella phaffii*			x				
*Trichophyton violaceum*		˅	x	*Lunulospora curvula*	x	X					
*Uncultured Nectriaceae clone*			˄	*Meyerozyma guilliermondii*	X	X	X				
*Ustilaginoidea virens*			X	*Microdochium* sp.	X						
				*Naganishia albida*	˅						
				*Naganishia diffluens*	X						
				*Penicillium chrysogenum*	X	x					
				*Penicillium griseoroseum*		X					
				*Penicillium olsonii*		X					
				*Pichia fermentans*	x	X					
				*Pichia guilliermondii*	x	X					
				*Pithomyces chartarum*	X						
				*Rhodotorula glutinis*	X	X	X				
				*Rhodotorula mucilaginosa*	X						
				*Talaromyces purpureogenus*	X	x					
				*Trametes hirsuta*		X					
				*Uncultured fungus*	˄						
				*Ustilaginoidea virens*	X	X	X				

## Data Availability

The sequences of the obtained species have been deposited with GenBank—links are provided in Table 2, https://www.ncbi.nlm.nih.gov/nuccore/.
